# Letter to the Editor in response to the forgotten prisoners: diet and behaviour in carceral systems

**DOI:** 10.1017/S0007114524001508

**Published:** 2024-08-28

**Authors:** Matthew Poulter, Shelly Coe, Catherine Anna-Marie Graham, Bethan Leach, Jonathan Tammam

**Affiliations:** 1 Centre for Nutrition and Health, Faculty of Health & Life Sciences, Oxford Brookes University, Oxford OX3 0BP, UK; 2 Cereneo Foundation, Center for Interdisciplinary Research (CEFIR), Vitznau 6354, Switzerland; 3 Lake Lucerne Institute, Vitznau 6354, Switzerland; 4 Practice Plus Group, Hawker House 5-6 Napier Court, Napier Rd, Berkshire RG1 8BW, UK

## Dear Editors,

Thank you for the opportunity to respond to insightful commentary on our systematic review^(1)^ on dietary interventions and the behaviour of prisoners. It is gratifying to see that our review is generating interest soon after publication.

We are aware of the ground-breaking seminal work produced by Schoenthaler and colleagues and are grateful for the time taken to respond to our review. We agree that research in this field within this population group is often overlooked and that further discussion around this subject matter is important.

We note the concern raised regarding our exclusion criteria, specifically our decision to include only studies with a control or the comparator group. Our intention was to ensure methodological rigour and consistency, aiming to synthesise evidence from studies with clearly defined control measures. This approach was chosen to enhance the reliability and validity of the findings by reducing potential biases and confounding factors. This did therefore lead to the exclusion of studies referenced in your letter, which involve quasi-experimental time-series designs, but we acknowledge the value of these formative studies to offer insights into the potential effects of dietary interventions on behaviour within correctional facilities.

Moreover, the transformation of dietary policies in the Maine carceral system highlights significant research opportunities for investigating the impact of dietary interventions on behaviour and mental health. We agree that such investigations could contribute to multidisciplinary research efforts and facilitate a deeper understanding of the relationship between diet and prisoner health. Indeed our research group has recently received a UKRI KTP award to investigate the impact of a nutrition educational programme on the diet of UK prisoners, with important implications for both health and policy change^(2)^.

We appreciate the opportunity to engage in constructive dialogue and remain committed to advancing the science of diet, behaviour and mental health in incarcerated population groups through rigorous research and collaboration, and your letter highlights further avenues for future exploration.
